# States, global power and access to medicines: a comparative case study of China, India and the United States, 2000–2019

**DOI:** 10.1186/s12992-024-01092-2

**Published:** 2025-02-01

**Authors:** Berit S. H. Hembre, Maulik Chokshi, Steven J. Hoffman, Fatima Suleman, Steinar Andresen, Kristin Sandberg, John-Arne Røttingen

**Affiliations:** 1 Norwegian Institute of Public Health, Postboks 222 Skøyen, Skøyen, Oslo, 0213 Norway; 2 Institue of Health and Society, Faculty of Medicine, University of Oslo, PB 1130, Blindern, Oslo 0318 Norway; 3 Norwegian Medical Products Agency, PO Box 240, Skøyen, Oslo 0213 Norway; 4 Blavatnik School of Government, Radcliffe Observatory Quarter, University of Oxford, Woodstock Road, Oxford, OX2 6GG UK; 5 ACCESS Health International, New Delhi, India; 6 Global Strategy Lab, York University, 4700 Keele Street, Toronto, ON M3J 1P3 Canada; 7 Wellcome Trust, 215 Euston Road, London, NW1 2BE United Kingdom; 8 School of Health Sciences, University of Kwa-Zulu-Natal, Private Bag X54001, Durban, 4000 South Africa; 9 Fridtjof Nansen Institute, P.O.Box 326, Oslo, 1326 Norway

**Keywords:** Access to medicines, China, India, United States, Structural power, International political economy, COVID, Vaccines, Emerging economies, Global health

## Abstract

**Background:**

What constitutes state`s global power to shape access to medicines? How was it distributed between states and how did this change from 2000 to 2019? In this comparative case study, we explored the powers of China, India and the United States, and discuss whether our findings from the pre-pandemic era were reflected in the global COVID-19 response related to pharmaceuticals. We used an analytical framework from the international relations literature on structural power, and assessed the following power structures after adapting them to the context of access to medicines: finance, production, financial protection, knowledge, trade and official development assistance.

**Results:**

We found that from 2000 to 2019 there had been a power-shift towards China and India in terms of finance and production of pharmaceuticals, and that in particular China had increased its powers regarding knowledge and financial protection and reimbursement. The United States remained powerful in terms of finance and knowledge. The data on trade and official development assistance indicate an increasingly powerful China also within these structures. During the COVID-19 pandemic, we found that the patterns from previous decades were continued in terms of cutting-edge innovation coming out of the United States. Trade restrictions from the United States and India contrasted our findings as well as the limited effective aid from the United States.

Building on our findings on structural powers, we argue that both structural power and political decisions shaped access to medical technologies during the COVID-19 pandemic. We also examined the roles and positions of the three states regarding developments in global health governance on the COVAX mechanism, the TRIPS Agreement waiver and the pandemic accord in this context.

**Conclusion:**

From 2000-2019, China and India increased their structural powers to shape global access to medical technologies. The recent COVID-19 pandemic demonstrated that both structural power and political decisions shaped global access to COVID-19 technologies.

## Background

 Access to medicines is a policy domain that transcends health, industry and trade of nations. Medicines are an integral part of any health system, and having access and availability of medicines may add trust in governments. 25 years after the Agreement on Trade-Related Aspects of Intellectual Property Rights (TRIPS) was signed, which is a legal agreement between the member states of the World Trade Organization (WTO), and after the HIV/AIDS epidemic put access to medicines on the global agenda, nearly 2 billion people still lacked access to essential medicines in 2018 causing preventable suffering [1–6]. During this period countries in Asia had gained power in financial terms, and in this study we wanted to explore the issue of access to medicines in the context of the geopolitical rise of China and India [7–10].

We see a case for focusing on the role of states. Both the United Nations (UN) High Level Panel on Access to Medicines and the Lancet Commission on Essential Medicines directed the majority of their recommendations towards governments [3, 11]. And both reports reflected the role of states in shaping access at home and abroad through a wide set of policies and interaction with other actors, including pharmaceutical companies and multilateral institutions. In the two decades prior to the COVID-19 pandemic, developments including globalization and shifts in governance regimes for medical research and development (R&D) led to a focus on transnational health policies and proliferation of actors beyond states in global health [12, 13]. The autonomy of states in shaping their policies related to access to medicines became a core issue though as it was limited by the WTO TRIPS agreement which extended intellectual property (IP) rights globally, especially supported by high-income countries hosting large pharmaceutical industries [14, 15]. Some states in the global South, including India, South Africa and Brazil, are well known examples within global health where states through policies and legal processes have emphasized public health in relation to IP and trade issues [16–19].

During the COVID-19 pandemic the key role of states in providing access to medical technologies and infection control was evident. In this study, we ask what constitutes state power when it comes to shaping access to medicines, and how power shifted between two emerging countries, China and India, in comparison to the traditional power of the United States of America (US) in the two decades before the COVID-19 pandemic. We describe if and how this power changed over two decades, and in the discussion reflect upon whether these findings were mirrored in the global response to access to pharmaceuticals during the COVID-19 pandemic, including the roles and positions of these three states regarding recent developments in global health governance in order to shed light on global politics of access post COVID-19.

Our study is set in the international relations (IR) and international political economy (IPE) literature on global health [20]. This study was conducted as part of a research project exploring the roles of China and India in shaping global access to medicines.

The US was chosen as a third case as we wanted to compare the two emerging powers to the main single state power. We consider these three states particularly relevant to global health due to the size of their populations and economies. We include both indicators on financial protection and reimbursement regarding domestic populations, as well as official development assistance (ODA) and foreign aid for health, as both may be relevant to global access of medicines and contribute to countries making progress on achieving universal health coverage (UHC).

In the past decade, several global health studies rooted in IR on China and India were conducted as part of studies of the BRICS (Brazil, Russia, India, China and South Africa). The main question of these studies included how these increasingly wealthy countries would contribute to global health through shaping the agenda, providing development aid, assessment of progress towards UHC and also more specifically through providing access to medicines [21–25]. The BRICS have diverged in their economic performances, but in recent years gained geopolitical importance as a block and has included additional members, and the rise of both India and China remains main stories in IR in our time [26, 27]. Notably, the role of China in terms of its foreign engagement has gained attention regarding if and how it differs from that of Western countries in relation to low- and middle-income countries (LMICs) in the Global South [28].

The global health discourse on access to medicines has developed over time. In this paper we study the pre-COVID era 2000–2019, and also explore the roles of China, India and the US in shaping access to medical technologies relevant to COVID-19. In the 1990s and 2000s the main focus of the access debate was on antiretroviral therpies for people living with HIV in developing countries [17]. Substantial progress was made in this area due to a complex set of actions, including an evolution of the interpretation and use of the legal framework on IP, India´s generic industry, as well as donations from HICs, notably the US [17, 29, 30]. In the past decade new and expensive medicines, including treatments for tuberculosis, hepatitis, cancers and neurological diseases gained attention, and access to medicines has become a part of the domestic health discourse in both low-, middle- and also high-income countries [3]. Pricing of pharmaceuticals, and a rise in inequality not only between countries but also within countries, contributed to this universal attention to the access debate [31, 32]. Scholars Moon and t`Hoen framed this shift in the 2010s as the global politics of access to medicines transformed “from 1.0 to 2.0” [33]. Following COVID-19, pared with increased geopolitical tension, the politics of access to medicines may have entered a new phase, and in the discussion we explore the positions held by China, India and the US in global health governance processes related to access to medicines after 2019.

As access to innovative medicines is scarce due to high drug prices and regulatory challenges affecting markets, and innovation of much needed new drugs is even scarce due to high costs of R&D and lack of market incentives for several health needs [34]. Access to off-patent drugs may be limited by affordability and adequate supply [35]. The access debate is relevant to several other major issues within global health, like health security, social health protection, and antimicrobial resistance [36, 37]. The 2019 Coronavirus outbreak as well as dissonant views on trade and trade competition between the United States and China attracted attention to the issue of access to medical products and state power, and put pharmaceutical supply chains on the top of the global agenda [38–42].

In the global health literature, the main contributions of China, India and the US in shaping access to medicines prior to the COVID pandemic have included the following:

The US was a major innovator of medicines, and had been pushing for strict intellectual property (IP) protection in trade agreements, and had at the same time been a major donor of foreign aid targeted for access to medicines and vaccines [43]. As mentioned, the issue of IP became central to the access debate related to the World Trade Organization`s TRIPS agreement, which was signed in 1994, and which according to Susan Sell “reduced polity making autonomy in intellectual property” [15]. She pointed to the central role of US based transnational cooperation’s in shaping TRIPS, but also put politics at the center of IP issues describing how especially US state power mattered in relation to both TRIPS and bilateral trade negotiations on IP protection [44].

India had been engaged in the global debate and key negotiations on access, and it´s massive generic exports in addition to an expanding vaccine industry had earned the country the reputation of being “the pharmacy of the South”, enhancing access to medicines, especially in LMICs [45, 46]. Generic production of antiretrovirals for people living with HIV/AIDS in India was possible because the Indian Patents Act did not provide for patents on pharmaceutical products until required by TRIPS in 2005 [47]. Many countries could import generic ARVs, largely because India could produce and export them. There was great concern in the public health community regarding access to medicines when India had to begin granting pharmaceutical patents under its TRIPS obligations [48, 49].

China has been engaged in technical medical assistance since the 1960s [50]. It´s industry had become world leading in exports of active pharmaceutical ingredients (APIs), and in the past decades China reformed it´s health system with access to medicines as one of the main focus areas domestically [51, 52]. China received significantly less attention than the US and India in the discourse on access to medicines and IP especially before the 2010s. Both academic works and news articles addressing the profile of the industry in terms of structure (fragmented) and level of innovation (from mainly generic to increasingly innovative) as well as China`s pharmaceutical policy and IP system have emerged [53–56].

We start out by presenting the study design, the theoretical framework of structural power and data sources. In the findings section we present data according to power structures after a brief introduction on demography and governance. In the discussion we summarize key findings, discuss if they were mirrored during the recent pandemic and also discuss the roles and positions of China, India and the United States in some of the global health governance processes following COVID-19. We discuss implications for global public health and suggest future research.

## Materials and methods

### Conceptual tools

To answer the research questions we have taken the following four steps: First, we adopted a theoretical framework on structural power by Susan Strange to the sectoral level of access to medicines [57]. For Strange, analysis of international political economy (IPE) should always be rooted in the sectoral level, which should inform the more general analysis [58]. McInnes and Lee applied Strange for an analysis of the issue of access to medicines in their 2013 book [20].

Second, we identified indicators to conceptualize and assess each power structure, based on a scoping review on the issue of access to medicines in the global health and political science literature as part of a research project on the roles of China and India in shaping global access which included this paper. For an overview, see Table 1. In addition to Strange`s power structures, we also include a brief section on key features of the three states on economic systems, forms of government and demography. Third, we identified what data were available for each indicator to shed light on the broader research questions. The fourth step of our analysis was to sum up findings, and discuss them in light of developments following the COVID-19 pandemic in terms of access to medical technologies and global health governance, as well as implications for domestic and global access to medicines.

**Table 1:** Power structures and indicators

***Power structures***	*Indicators*
**Finance**	• Gross Domestic Product (GDP) • Health care spending
**Financial protection and reimbursement: ** **(Strange`s security** **structure)**	• Insurance coverage • Out of pocket expenditure and catastrophic health expenditure
**Production **	• Gross production • Profile of production: Active pharmaceutical ingredients, formulations, biologics • Ownership of industry
**Knowledge**	• Input: Investment from states in research and development • Output: Publications, patents, bringing new drugs to the market
**Trade:**	• Value of imports and exports of pharmaceuticals • Trading partners • Key developments regarding trade and intellectual property issues
**Official development assistance** **(Strange`s welfare structure) **	• Official development assistance

### Theoretical framework: structural power

“Structural power, in short, confers the power to decide how things shall be done, to shape frameworks within which states relate to each other, relate to people, or relate to corporate enterprises” [57]. Strange identified four key interrelated primary structures underpinning IPE: Production, finance, knowledge and security, as well as secondary structures including trade and welfare, which depend on the primary. We have left out the secondary structures of transport systems and energy, as they can be regarded as more peripheral to the issue of access to medicines in the global health discourse and due to the size of the study (Tables 2, 3, 4, 5, 6 and 7).

A key question for Strange is *who benefits*, which sits well with the question for the discussion on who gets access [58]. Strange argued that to look at who benefits you need to know where the power lies and how this influences outcomes, in line with the realist school of IR according to Christopher May [58].

The financial structure according to Strange mainly concerns the international monetary system and international finance and debt. The economic power shift towards Asia and its potential implications for access to medicines motivated this study. For this power structure we explored shifts in Gross Domestic Products (GDP) and healthcare spendingr, which overlap with the security structure.

The security structure is the framework of power created by the provision of security by some human beings for others. Here it is important to highlight that we did not apply the concept of health security. We attended to how states may provide security to citizens through provision of health care and access to medicines through financial protection and reimbursement [59]. We will return to definitions of security in the discussion. A main indicator is out of pocket expenditure (OOP) and catastrophic health expenditure. We also included insurance coverage, and the share of public plans.

The production structure concerns who produces what, where and under what conditions. From an access to medicines perspective, this can be linked to the size and profile of pharmaceutical production in the countries, and ownership of these pharmaceutical companies.

The knowledge structure is about conveying knowledge, and more broadly about what is known and believed, and the infrastructure through which this is communicated. Pharmaceutical innovation emerges out of an interplay between states and industries. Regarding *input*, we assessed investments into R&D. On *output*, we assessed capacities for R&D in terms of scientific publications, patents and bringing new drugs to the market.

For Strange, power also lies in the capacity to deny knowledge, and here, we see overlaps to the secondary structure of trade, and transfer of technology, which is significant to the discourse on access to medicines [1]. The structure also involves what is sold, to who and on what terms. On trade, we explore major trends in these states´ policies related to trade and intellectual property rights (IPR) on pharmaceuticals, as well as imports, exports and major trading partners.

On the so-called welfare structure, Strange frames welfare in the context of IPE and writes that “there is more to welfare in the context of the global political economy than the foreign `aid`”. And she points to that that there are many forms of resource transfer, and also that aid may not differ much from loans at commercial rates, and may be motivated by self-interest. In the field of global health though foreign aid has held a role in shaping access to medicine, and we focus on the official development assistance provided by the three states.

### Case selection

This paper was written as part of a research project on the roles of China and India in shaping global access. We wanted to compare the two to a third case, a traditional and major pharmaceutical power. We considered including the EU, but decided to focus on the US which until the COVID-19 pandemic was the main single state power. We decided on the US though due to the following aspects: First, the US, like China and India, is a major economy, houses a large population and domestic market as well as needs, and have a substantial pharmaceutical production. Second, the US and India have presented diverging views on IP issues related to access to medicines over decades, and thus the two could represent a frame for discussing the position of China. Third, as we started on this project at a time where great power rivalry was escalating, especially between China and the US, and assessing their powers in the field of access to medicines seemed interesting, and also relevant as the COVID-19 pandemic spread. We also considered including more of the BRICS, South Africa and Brazil, but due to administrative and capacity limitations we were not able to do so.

### Data

The starting point is set to 2000, chosen from a deliberate and practical perspective, as it was the year before China entered the World Trade Organization, and earlier data are difficult to obtain [60]. The data collection took place from 2018 to 2019, and thus mirror what was known prior to the COVID-19 outbreak.

### Findings

#### Key features of the States

According to the WTO both India and the US were market economies, whereas it was disputed whether China was [61]. India and the United States were the world´s two largest democracies, in China the Communist Party was the sole party of power. All three states had a similar share of the population aged 14–65 years (ranging from 65 to 71% in 2018), but India had a younger population than China and the US [62]. The population aged 65 and above was 6% in India compared to 16% in the US and 11% in China in 2018.

#### Finance

From 2000 to 2019 the GDP growth in China and India exceeded that of the US, whereas the US had the world`s highest GDP and also a significantly higher GDP per capita [4, 63]. The US spent more on healthcare than any country, and had high per capita expenditure compared to most HICs and a high share of GDP was spent on health [64]. The government expenditure on health in India was low compared to other lower-middle-income countries and even compared to the average for low-income countries [65]. India saw a decrease in GDP spent on health from 4% in 2000 to less than 3% in 2018. China had seen an increase in the share of GDP spent on health, from about 4,5% to 5,7% from 2000 to 2018, which was higher than the 5% that was indicated as a minimum target for countries by the WHO and a 2014 Chatham House report [66–68].

**Table 2:** Finance

	USA	China	India	Major differences	Sources
**GDP ranking**	Largest GDP of any state 2000–2019.	In 2000 the 6th largest economy by GDP, since 2010 the 2nd largest GDP.	In 2000 ranked as the 13th and in 2018 the 6th largest GDP.	The US GDP in 2018 was 6 times that of China and 31 times India`s.	[4] [63] [64] [65] [69] [70] [71] [72] [73] [74]
**GDP increase, 2000-18**	GDP doubled, 10,285–20,544 bn USD.	A 10-fold increase, 1211–13,608 bn USD.	A 6-time increase, 462–2719 bn USD.		
**Health care spending**	1st 2000–2017, increased from 58 to 117 bn current USD.	In 2000 ranked 14th and 2nd in 2016.	In 2000 ranked 17th and 7th in 2016.		
**Percentage of GDP spent on health, 2000-17**	12.5–17.1%	4.4–5.3%.	4.0% − 3.5%		
**Per capita spending on health, 2000-17**	4560–10,246 USD	42–393 USD	19–59 USD		
**Domestic government health expenditure 2000-17, share of public spending**	5.5% − 8.6 In 2014, public spending accounted for 49% of total.	1.0 − 2.9%. In 2017 a half of current healthcare funding was financed by public sources.	0.8 - 1% About 30% of all healthcare was publicly funded in 2014.		
**Pharmaceutical spending per capita, 2015**	1177 USD	333 USD	23 USD		

#### Financial protection and reimbursement (Strange`s security structure)

In terms of health insurance, the introduction of the Universal Medical Insurance System in China in 2008 stands out as it led to a massive increase of the coverage rate from 50% to 97,5% by 2014, and also some increase in availability and use of medical services [75, 76]. In comparison, the 2014 US Affordable Care Act reduced the percentage of un-covered by 4,5% points 2013-17, but the proportion uninsured increased from 2017 to 18 [77, 78]. Coverage in India remained low at about 20%, but in 2018 the government announced a reform which was set to expand access to healthcare for nearly half of the population through tax supported health insurance scheme Pradhan Mantri Jan Arogya Yojana, which showed some positive results in decreasing out of pocket expenditure, though there were worries regarding limited sustainable financing [79, 80].

Out of pocket expenditure as percentage of current health expenditure fell from 2000 to 2015 in all three countries [81]. OOPs per capita (PPP, current international dollar) increased in all three countries from 2000 to 2018, and the most in China, India had a peak in 2013-16 [82]. The incidence of catastrophic health expenditure was significantly lower in the US compared to both China and India [74]. Himmelstein et al., pointed out though that 62% of all personal bankruptcies in the US in 2007 were medical, and that medical impoverishment is rare in HICs save the US [83].

**Table 3:** Financial protection and reimbursement (Strange`s security structure)

	USA	China	India	Major differences	Sources
Coverage, including both public and private insurance schemes	Increased from 85–91% 2008-17, 2017-18 there was decrease.	Strong increase from 50–97% 2005-11, and stable rates thereafter.	Relatively low coverage, about 20–33% of the population.	China saw a massive increase in coverage rate. The US remained its high insurance coverage rate, and India its low rate.	[81] [82] [80] [75–77, 84] [85] [86]
Share of public plans (% population)	Increased from 27–38% (2005-17). The share with private insurance remained relatively stable.	In 2018 more than 95% were covered by basic public health insurance. In 2018 private schemes accounted for 14% of the total insurance premium income.	Both private and public schemes were available.		
Out of pocket spending as percentage of current health expenditure: 2000-15	15 − 11%	60 − 32%	72 − 65%	The US levels of OOPs and catastrophic health spending were significantly lower than China and India`s.	
Incidence of catastrophic payment: In the latest year available 2006–2015 (Year not specified, on the 25% threshold of household consumption)	0.8%	4.8%	3.9%		

#### Knowledge

We did not find recent data allowing for direct comparisons of public or private investments in pharmaceutical R&D, but rather data suggesting declined US research funding relative to increased Chinese. A similar trend was found regarding patents and scientific publications where China surged, and India saw a more modest development [87, 88].

On the capacity to bring new drugs to the market, no companies based in China or India held the patent of top-10-selling drugs globally (by revenue) in 2019, or got novel drug approvals by the US FDA 2016–2018 and thus access to the world`s largest pharmaceutical market [89, 90]. A study which evaluated the dominance of the US in pharmaceutical innovation examined core patents covering new drugs approved by the US FDA 1996–2010 demonstrated that the US was still dominating in the innovation network, especially when it came to essential core inventions [91]. Bearing in mind the long development timelines of 10–15 years, uncertainty of new drugs discovery, and regulatory hurdles, scholars argued that it still might have been too early to find major shifts in emerging economies footprint on global drug innovation [92].

There is little doubt, though, that significant pharmaceutical R&D was taking place in China and India. The China homegrown cancer-drug fruquintinib can serve as an example to illustrate potential increased innovation capacity [93]. Chinese FDA approved the drug in 2018 and the company was attempting to access the US market [94]. One example of R&D significant to global health is the work done in India on developing pediatric formulations of HIV drugs [95].

**Table 4:** Knowledge

	USA	China	India	Major differences	Sources
**Gross domestic expenditure on R&D (GERD) from 2010 or latest year (before 2013)** **Share of total GERD spent on health**	119 bn PPP $ 29%.	9 bn PPP $ 4.9%	5 bn PPP $ 16.6%	Findings indicate increased Chinese investments into pharmaceutical R&D, but the US remained leading.	[52] [87] [88] [89] [90] [96] [97] [98, 99] [100, 101]
**Publications in life sciences, global ranking, 2019**	Ranked 1st	Ranked 4th	Ranked 23rd	The US remained leading in terms of number and top cited publications. China climbed publications rankings substantially.	
**Share of 1% most cited science academic publications,** **2000-14**	62% − 55%	2–11% In 2014 s only to the US and the UK.	Less than 4% in both years.		
**Patents filed in country patent office, ranking, 2004-18**	360 –600 K Until 2011 the US received the most applications, and until 2019 was the top source of international patent applications, in 2019 the US was second in both respects.	130 K − 1,5 Million In 2011 China´s patent office became world leading measured by the number of patent applications received. In 2019 China surpassed the US as the top source of international patent applications.	17 –50 K India was ranked 7th in terms number of patents filed in 2018.	Patent filing increased by 12 times in China, 3 times in India and 1, 7 times in the US 2004–2018. China surpassed the US in patent filing.	
**Pharmaceutical patents counted by applications origin,** **2000-18**	5308 - 11,931	1220–7362	29–393.	On pharmaceutical patent grants the US remained leading.	
**FDA Novel Drug Approvals**	Housed 60% of headquarters of companies granted novel drug approvals by the FDA 2016-18.	None	None	The US housed the majority of companies with global top-selling drugs, as well as those receiving novel drug approvals from the FDA.	
**Top selling drugs**	Of the 10 top-selling drugs (revenue) of 2019, 8 had their patents held by companies with headquarters in the US.	None	None		

#### Production

For this power structure it was challenging to find comparable data on the value and volumes of total pharmaceutical production from publicly available sources, and thus it was difficult to compare between the cases. We have found data suggesting the size of pharmaceutical productions, but that we are not sure if comparable [102–104]. These data are in line with other sources reporting that the US held a leading position in the global pharmaceutical market followed by Europe and then China at the end of the period our study covers [105]. According to a 2021 report though, by 2014 China had the world`s largest, the US second and India 12th largest production in value, but one obstacle when determining the scale of pharmaceutical production and exports is that some APIs might be used in more industries than the pharmaceutical [106, 107]. China became world leading in terms of API production and India produced the most formulations [52, 108–110]. The majority of the most profitable pharma companies were based in the US [111]. All three countries housed biological industries, of which the production is more complex and costly than producing chemical entities [72, 109, 112].

In terms of ownership of the industry, we highlight some key developments regarding openness to foreign investments and state ownership. The U.S. placed limitations on foreign investments in the pharmaceutical industry, due to concerns over Chinese and other foreign investments in companies with advanced technology. The Committee on Foreign Investments was in 2018 given increased powers to limit overseas investments in strategically sensitive sectors, which might have reduced biotech investments especially from Chinese firms [113].

China´s transition into a market-oriented model has also been playing out in the pharmaceutical sector, and the Chinese pharmaceutical industry was opened up to foreign investments prior to the period studied here [114]. And about 30% of pharma companies had a degree of foreign ownership according to a 2017 study, but some large companies remained state-owned, including Sinopharm [55, 108].

Indian pharmaceutical companies have largely been family-controlled, but mergers and acquisitions by foreign firms might be changing that, the industry was opened up for foreign investments in the 1990s [115, 116].

**Table 5:** Production

	USA	China	India	Major differences	Sources
**Gross production in USD in 2014, share of own input goods (in parenthesis)**	213 bn (45%)	304 bn (70%)	20 bn (61%)	The US and China had larger gross productions than India in value, the US was the least self-sufficient in terms of input.	[52] [112] [107] [111] [113]. [108] [109] [117]
**Ownership**	In 2019, 6 of the top 10 pharmaceutical companies according to their revenue were based in the US.	None	None	The majority of the most profitable pharma companies were based in the US.	

#### Trade

India had a positive balance of trade in pharmaceuticals in contrast to the US and China from 2014 to 18 [118].

In terms of access to medicines and trade in pharmaceuticals, IPR is a central issue, and the US and other developed countries with innovative pharmaceutical industries have pushed for strong IP protection in trade negotiations [1].

When joining the WTO in 2001 China extended all patent coverage to twenty years, and accepted US demands for “TRIPS plus provisions” [119]. China did not use the flexibilities offered in the WTO TRIPS regime and in the Doha Declaration, and issued no compulsory licenses. More recently China took steps towards stronger IP protection, which may benefit foreign companies at the cost of domestic generic industry in the short term, and contribute to building a more innovative industry in the long term [53, 56].

India had no pharmaceutical product patent protection from 1970 to 2005, which contributed to India becoming the “the pharmacy of the South/ world” due to massive generic production and exports to developing countries [29]. Despite the full implementation of TRIPS from 2005, India maintained its role as a key generic exporter though, through a complex set of policies, legal processes and licensing practices [115]. However, bilateral trade agreements may change the situation, within Indian companies needing to abide to stronger patent regulations. The US has been pushing India on pharmaceutical IP protection through trade negotiations [120]. Parallel to the global health success story of Indian pharma in terms of access in LMICs, trade deals have also opened up developed pharmaceutical markets to India. About 1 billion prescriptions in the US were fulfilled with drugs from companies based in India in 2018 [121].

**Table 6:** Trade

	USA	China	India	Differences	Sources
Imports: Share of global imports of pharmaceuticals, in value, 2018 Annual growth of imports, 2014-17	7% 11%	4.9 13%	0.3% 6%	In 2018 the US was the major, China the 6th and India the 42nd largest importer of pharmaceuticals. The import-growth was higher to China than to the US and India (2014-17).	[106] [122] [123]
Exports: Share of global exports of pharmaceuticals in value, 2018 Ranking of global exporters of pharmaceuticals, in value, 2017	8.3%. 1% 4th	1.5% 16th	2, 4% 10th	The US was the 4th, China 14th and India 11th major exporter of pharmaceuticals in terms of value in 2018.	
Trading partners, 2018	Of top 10 exporting partners, 9 were HICs	Of top 10 exporting partners, 9 were HICs	Of top 10 exporting partners, 5 were HICs and 5 MICs	The US and China mainly traded with HICs whereas India included more MICs.	

#### Official development assistance from China, India and the US (Strange`s welfare structure)

The US remained the major donor to global health programs 2000–2019, and large programs targeted access to medicines where HIV alone made up about 50% of the budget [124, 125] .

A paper by Tang et al. estimated China´s donations to health aid in 2013 to 7 US Billion [126]. Chinese aid to Africa has gained attention, including questions on whether it confirmed to the OECD`s conceptions of developing aid standards [50, 127]. In 2018 the China International Development Cooperation Agency was established, which might contribute to increased transparency and coordination [128, 129]. The agency was involved in the Belt and Road Initiative, at core an infrastructure project, but with links to domestic and global health issues [130].

From the early 2000s India defined itself as a donor rather than a recipient, though our data found that India was a net receiver also in 2017 [131]. Steps were taken in the 2010s towards improved coordination and centralization of its outward aid [128].

**Table 7:** Official development assistance (Strange`s welfare structure)

	USA	China	India	Major differences	Sources
Official development assistance (ODA):	The biggest donor of ODA. ODA increased by 344% to 34.4 bn USD from 2000–2016. In 2019 the US was the leading donor to global health programs, donating 11 bn USD.	A net receiver of ODA in 2000. By 2011 a net donor, in 2017 the net ODA donation was 1.05 bn USD.	A net receiver of ODA in 2000. In 2017 the net ODA received by India was 3,09 bn USD.	The US donated almost 33 times that of China in ODA in 2016.	[124]. [132] [133].

## Discussion

### Principle findings on structural powers to shape global access to medicines, 2000-19

Decades into “the rise of Asia”, it was notably that the US remained dominant along most power structures. It remained the largest economy. It spent the most on health, and compared to China and India provided better financial protection, but not compared to most HICs. It housed the majority of innovative firms that brought the most profitable new medicines to the market, and the US was also the largest contributor to R&D for neglected diseases. The US had been key in shaping the global trading system, including protection of IPR. In terms of health aid, the US remained by far the main contributor despite internal political disputes on the topic.

China had become the second largest economy by nominal GDP, the largest in the world by purchasing power parity, and a leading producer of pharmaceuticals. It had expanded health insurance coverage through public schemes. Both the US and India had been concerned regarding their dependency on Chinese API production. New and innovative drugs seemed increasingly to be coming out of China and the trade and IP system was being transformed to serve innovative industries. China increased its investments in foreign aid, and set up China-led institutions.

India had also seen massive economic growth, but health insurance coverage rates remained low, and public healthcare spending was low, also when compared to LICs. India was a key exporter of generics, both to LMICs and HICs, and more so to LMICs than the US and China. Generic production and exports were maintained also after TRIPS, in part due to public health safeguards being included in the Indian Patents Act. India remained both a recipient but also increased its donations of foreign aid.

### The findings discussed in the light of the Covid-19 pandemic response: Developments, data

Were these findings mirrored in the global response to access to medical technologies during the COVID-pandemic? We would suggest so in terms of knowledge/R&D and in part production, but see more divergence on trade and aid, which we will elaborate on, before discussing the relevance of how both structural power and political decisions determined global access to medical technologies during the recent pandemic.

Access to pharmaceuticals, like effective drugs for treating COVID-19 and vaccines for prevention, became key in the pandemic response as states sought to protect their citizens. Innovation for both new and older treatments came out of the US as well as cutting-edge mRNA technology for vaccines [134, 135]. Both Indian and Chinese firms licensed US technology to produce antivirals, through bilateral agreements or the Medicines Patent Pool [136], but manufacturing was not scaled up due to a complex set of reasons [137]. China produced homegrown drugs [138]. Indian and Chinese R&D led to COVID-19 vaccines based on established technologies, in particular inactivated viral vaccines [139]. Notably, in the earlier phase of the pandemic by October 2021, China had exported more vaccines than all other states combined [140].

### Structural power and political decisions

Ronan Polan pointed out that Strange argued that actors may *have* structural power, but also that “government`s structural power can be discerned in decisions and non-decisions” [57]. There are different ways to define and frame political power [141, 142]. Susan Sell in her 2001 paper on the TRIPS campaign, state power and agency wrote that her “perspective endeavors to put politics at the center of intellectual property issues. Who gets what, when and why?” [15] Which is related to Strange`s key question in States and Markets: Who benefits? In States and Markets Strange also describes how she understands *welfare* in the context of International Political Economy, namely that allocation of welfare among states is dependent on both the primary power structures, but also “the uses to which it is put” [57].

During and after the coronavirus pandemic global health scholars and others have written extensively on vaccine diplomacy and “vaccine apartheid”, the COVAX mechanism for vaccine distribution, the TRIPS waivers for vaccines and for diagnostics and therapeutics, the pandemic accord and the role of politics of different states in these [143–146]. Here we consider some aspects related to China, India and the US, on how they used their capacities in the pharmaceutical domain as well as political power to shape global access to medical technologies during the COVID-19 pandemic and in developing global health governance regimes.

The COVAX mechanism as a part of the Access to COVID-19 Tools Accelerator which included GAVI, CEPI, the WHO and UNICEF, received substantial criticism for delivering vaccines late [143, 144]. A main criticism directed towards HICs including the US was that they set up bilateral agreements with companies, and thus undermined COVAX multilateral agreements [144, 147]. The US, like most states, prioritized supplying its own citizens with drugs and vaccines [148]. According to some, COVAX`s efforts were initially primarily constrained by a lack of vaccine supply rather than a lack of funding, whereas others highlight that early funding would have made COVAX better able to secure supply by entering into contracts alongside HICs governments [149]. China, India and the US joined COVAX. While China in 2021 had become a key supplier of vaccines globally, the US was the largest donor to COVAX [140, 144]. However, China later struggled to ease domestic lockdowns due to lower vaccine efficacy of the Chinese manufactured vaccines and lower uptake than in many other countries [140, 150]. China mainly sold rather than donated vaccines, including to COVAX [144]. China was also a key global source for protective equipment and COVID diagnostics [140, 151]. Both motives and outcomes of China`s “mask diplomacy” and “vaccine diplomacy” have been widely studied and analyses include motives like soft power and economic opportunities [152, 153].

In contrast with our findings pre-COVID-19, limited exports of vital ingredients from the US to India and export restrictions on vaccines from India may have hampered the COVAX mechanism that relied heavily on Indian production capacity, perhaps limiting the effectiveness of vaccine aid [154–156]. India`s export restrictions were introduced as its domestic COVID-19 cases spiked during the delta-wave.

To sum up, we suggest that structural power, and especially capacity for R&D and production as described in our findings, were key to China`s vaccine diplomacy. However, also political decisions led to China being a main exporter of vaccines to low- and middle-income countries. Regarding the US, shifting administrations had diverging attitudes towards global health initiatives, and President Trump in 2020 announced that the US would halt its relationship to the World Health Organization, which was a decision also linked to US-China relations [157]. Despite continuing its ODA commitments, these developments may have weakened the US positions in terms of global health leadership [158].

Whereas Susan Sell pointed out the key role of American based transnational corporations in shaping TRIPS through lobbying towards the US government on intellectual property rights and enforcement, COVID-19 also highlighted complex interactions between states and industry. In the US context, both technology challenges from China as well as previous extensive offshoring of pharmaceutical production especially to China and India, was part of the backdrop for Operation Warp Speed (OWS) [159]. OWS was a public-private partnership set up to provide medical technologies related to COVID-19 [160]. Some see it as a first of a series of US major industrial innovation policies, potentially inspired by Chines funding for industrial scale-up, though concerns have been raised regarding whether it allowed for pharmaceutical companies to take advantage of the COVID-19 crisis [159, 161]. On the geopolitical dimension of OWS, Kim et al. in 2021 wrote the following in the Lancet: “Recipients of OWS funding also have clear commitments: to the USA. Companies that are supported by OWS, and manufacturers in Russia and China, have approached countries and organizations independently, creating a complicated ecosystem for COVID-19 vaccines that is comprised of a patchwork of countries that have and do not have vaccines” [162].

The issues of IP and trade, have also been at the center of the discourse on access to medical technologies during and following the COVID-19 pandemic. India and South Africa proposed the patent waiver in the WTO TRIPS Agreement, calling for a temporary patent waiver for COVID-19 drugs, vaccines and related equipment technologies. This received massive attention and may shed some light on the positions of the states in question. India`s position seems to have been in line with that of previous years, promoting public health interests over IPR and thus also its own generic industry that could benefit. The US first opposed the waiver, then turned with the new Biden administration, but limited it to vaccines. The US, together with the EU, which initially did not see IPR issues as a barrier to access to COVID-19 technologies, alongside India and South Africa, negotiated a compromise [163]. China was “neither a proponent nor a cosponsor of the waiver proposal”, but it supported the initiative according to Peter Yu [164]. He framed this as a middle-of-the-road position in line with what Jeremy Youde described as ambiguous positioning in global health governance, and put China between the proponents and opponents of the waiver [165]. The vaccine waiver was approved by WTO in July 2022. However, later attempts to agree a waiver for other COVID-19 technologies have failed to ensure consensus. Haugen in 2021 asked if TRIPS prevented COVID-19 vaccines from becoming global public goods, and concluded that among pro-TRIPS developed countries (which included the US) there was an acknowledgement of obstacles by the IP system, “but their overall position on the IP system has not changed” [166].

### Implications for access to medicines and global public health, looking ahead

As China, India and the US possess structural powers for shaping access to medicines globally, both their structural powers and their policies will determine future access. The recent pandemic highlighted the vulnerability of global interdependency in times of shortages of medical products as well as political rivalry. Calls for domestication, “de-coupling” and “friendshoring” of production are currently present in many countries, which may impact the relative influence of these three states given that more distributed manufacturing capacity is likely to emerge. In terms of demography, population growth in this century is forecasted to mainly happen in LMICs with hitherto limited powers in the pharmaceutical domain [167]. Thus, how powerful states facilitate for access to medicines in times of diverging demographics and political rivalry will shape the lives of many.

There seem to be an increased capacity for pharmaceutical innovation in the two emerging countries studied, and thus outside OECD countries that traditionally have housed the innovative industry. Recent reports indicate that especially China further continues to increase its investments in pharmaceutical R&D [158, 168, 169]. More hubs for innovation may lead to new treatments and treatments for diseases also affecting the less affluent populations, i.e. neglected or poverty related diseases. The significance for global public health will depend on what diseases these medical technologies target and who will get access.

### Comparative strengths and weaknesses

Susan Strange presented her theory on Structural power decades ago as a response to the declinist school of American hegemony, to make the point that the finance structure, all though important, was not the single important parameter for assessing state power. This study takes this broad approach to understanding state power, which gives less room for in-depth analysis of the single structures or indicators. “Access to medicines” is a challenging concept to operationalize, and so is conceptualizing state power in this field, especially due to the complex interactions and many interfaces between states and markets. For some indicators good and comparable publicly available sources were scarce. We left out two secondary structures of infrastructure and energy, but these can be relevant for further studies [170]. Christopher May has pointed out that the knowledge structure may be the least defined and most problematic in Strange`s writing, and thus we could have included additional relevant indicators [58].

We gathered data for this study until 2020, and new knowledge has since emerged as the issues of state power and access to medical technologies gained massive attention due to the pandemic. One example is that the lack of data on the origins of APIs has been addressed, where some analyses have downplayed the reliance on China relative to India [171].

### Future research

A key question for Strange when developing her theory on structural power was: *Who benefits?* Going forward we would emphasize the need for research on how powerful states in the pharmaceutical domain can improve access to medicines for those in need living in states without such capacities, as championed by Indonesia during its 2022 G20 presidency, and also how they may decide to block or delay such access. If the model of innovation and aid mainly coming out of HICs and generic and API production to a large extend taking place in emerging MICs will be transformed, the global politics of access to medicines may be drastically altered, and the consequences especially for patients in lower MICs and LICs are indeed worth monitoring. Also, the incentive system for R&D may have changed due to challenges regarding access to medicines in HICs. The recent COVID-19 pandemic has increased the impetus for such a change and we believe such a transition may now happen faster. However, this also introduces risks, including related to quality of medicines due to regulatory capacities as well as human workforce capacities and capabilities.

## Conclusions

This study has taken a broad approach to understanding state power in the pharmaceutical domain, and explored the structural powers held by China, India and the US in the decades leading up to the COVID-19 pandemic. We found that all three states held substantial structural powers, and from 2000 to 2019 we found a power-shift towards both China and India especially along the structures of finance and production. However, the US remained powerful in terms of finance and knowledge. China increased its powers in particular regarding knowledge and financial protection.

During the coronavirus pandemic cutting-edge innovation continued to come out of the US. China was the key source of COVID-19 vaccines for low- and middle income countries in the earlier phase of the pandemic. Trade restrictions from the US and India during the COVID-19 pandemic contrasted our findings from the previous decades, as did the limited effects of aid due to supply constraints. Both the structural powers for R&D and pharmaceutical production as well as decisions to export vaccines made the Chinese “vaccine diplomacy” possible. Whereas the US produced more effective vaccines based on new platforms, and prioritized its own citizens, which may also have wakened the position of US global health leadership.

More hubs for innovation might lead to new diagnostics and treatments, but the significance for health depends on their effectiveness, what diseases they target and who gets access. The COVID-19 pandemic called attention to the vulnerability of global supply chains , but the potential consequences of “friendshoring” for global access are unclear and may be negative as globalization is slowing and protectionism is on the rise [172].

## Data Availability

All data generated or analyzed during this study are included in this manuscript and its references.
